# Incidences of Atonic Postpartum Hemorrhage and Related Risk Factors at a Tertiary Hospital in Saudi Arabia

**DOI:** 10.3390/nursrep10020020

**Published:** 2020-12-16

**Authors:** Wedad M. Almutairi

**Affiliations:** Maternity and Child Department, Faculty of Nursing, King Abdulaziz University, Jeddah 21551, Saudi Arabia; walmutairi@kau.edu.sa

**Keywords:** PPH, atonic PPH, risk factors, Saudi Arabia

## Abstract

*Background*: In 2017, there were 295,000 maternal deaths worldwide from preventable causes related to birth. The leading cause of maternal mortality is obstetric hemorrhage. In Saudi Arabia, a paucity of evidence about incidences of atonic Postpartum Hemorrhage (PPH) and related risk factors exists. Therefore, aims were to (a) identify incidences of atonic PPH from 2015 to 2018 (b) determine risk factors of atonic PPH in vaginal birth. *Methods*: Retrospective chart review with purposive sampling conducted revealed 386 charts, 220 (57%) vaginal birth and 166 (43%) caesarian section (CS). Logistic regression analysis was used. *Results*: Incidences of atonic PPH were 2.5% from 2015 to 2017, with the rate increasing by 12% from 2017 to 2018. In vaginal birth, significant associations between the severity of blood loss with epidural (F = 6.314, df = 1, *p* = 0.013), episiotomy (F = 4.38, df = 1, *p* = 0.038), induction of labor (IOL) (F = 1.224, df = 1, *p* = 0.004), and Interaction between IOL, AUG, and epidural (F = 7.24, df = 1, *p* = 0.041) found. *Discussion*: Increasing rate of atonic PPH confirmed. Epidural, episiotomy, induction of labor, and induction with augmentation are factors associated with severe atonic PPH in vaginal birth.

## 1. Introduction

An estimated 295,000 maternal deaths occurred worldwide in 2017, with 211 maternal deaths per 100,000 live births. The lifetime risk of maternal mortality is estimated at one in 190 births, the deaths in low-resource countries being 40 times higher than developed countries [1]. Every day in 2017, 810 women died from preventable causes related to pregnancy and birth [2]. Therefore, target 3.1 of the Sustainable Development Goals (SDG) of the United Nations is to reduce global maternal mortality rate (MMR) to less than 70 in 100,000 by 2030 [2] and to reduce the baseline rate of 2010 to 70% by 2030 [1]. Based on a 2017 World Health Organization (WHO) report, Saudi Arabia had an MMR of approximately 17%, and the reduction rate between 2000 and 2017 was only 29%. WHO, along with many organizations and international programs related to maternal health support, list strategies for ending preventable MM. One of the strategies is to determine the related causes of maternal death worldwide [1].

The leading cause of MM is obstetric hemorrhage [1]. Obstetric hemorrhage accounts for 27.1% of all maternal death worldwide [3]. Of all obstetric hemorrhages, postpartum hemorrhage (PPH) accounts for 72% [3]. Remarkably, PPH rates are rising worldwide [4,5,6]. Therefore, an important step to save mothers’ lives is to identify the magnitude of the PPH problem in each country by updating the evidence regarding incidences and causes of PPH in each country.

PPH is traditionally defined as blood loss greater than 500 mL in vaginal birth and greater than 1000 mL in caesarian section (CS) birth [7,8]. Primary PPH is defined as “bleeding from the genital tract of 500 mL or more in the first 24 h following delivery of the baby” [9]. In 2017, the American College of Obstetricians and Gynecologists defined PPH as cumulative blood loss ≥ 1000 mL or bleeding associated with signs/symptoms of hypovolemia within 24 h of the birth process regardless of delivery route [10]. Incidences of PPH have increased since 1994 even with medical preventive measures [10]. The factor most responsible for the high prevalence and increasing rate of PPH is uterine atony, that is, the failure of the uterus to contract efficiently after placenta delivery, accounting for more than 80% of PPH cases [11,12].

The associated risk factors of the primary atonic PPH are the following: multiple pregnancy, obesity (BMI > 30), advanced maternal age, first birth (nulliparity), history of PPH, and anemia. Risk factors related to pregnancy and the birthing process include gestational age, instrumental delivery, induction and augmentation of labor, prolonged labor (12 to <24 h), uterotonic use, macrosomia (large newborns), laceration, delivery by CS, and vaginal birth after CS [10,13].

WHO reports feature general statistics about trends in obstetric hemorrhage without concise information about incidences of PPH, causes, and the risk factors in each country. This offers a vital opportunity for researchers to investigate the incidences and causes of PPH in their populations. In Saudi Arabia, there is a paucity of evidence regarding incidences of the primary atonic PPH and its related risk factors. Therefore, the aim of this study is to (a) identify incidences of atonic PPH from 2012 to 2018 at a tertiary hospital in Jeddah, Saudi Arabia, and (b) determine the association of the severity of blood loss in atonic PPH women with the following risk factors: maternal age, history of CS, gestational age, birth weight (newborn weight), maternal body mass index (BMI), parity (number of pregnancies), episiotomy, induction of labor (IOL), and augmentation of labor (AUG).

## 2. Methods

We conducted a retrospective chart review study for women who delivered between January 2015 and December 2018 in a tertiary hospital in Jeddah, Saudi Arabia, with selected cases of atonic PPH. We defined cases as women with diagnoses of atonic PPH (International Classification of Disease ICD 10 code O721) chosen from hospital discharge records. Due to the inadequate adoption of ICD coding in the hospital, we also included the charts of women with documented estimated blood loss > 500 mL in SVD and >1000 mL in CS, excluding placental complications, coagulation dysfunction, uterine rupture in the labor and deliver registry book to ensure full access to the primary atonic PPH cases.

Information about the maternal characteristics, obstetric history, pregnancy, labor, and delivery was extracted from the medical records. Expert medical record abstractors used data collection sheets for each case, and inter-rater reliability was applied before data entry to SPSS software. The response variable was amount of blood loss in ml, while the continuous independent variables were age in years, BMI values, parity, gestational age, hemoglobin level at the time of birth, and birth weight. The categorical variables were having an epidural, episiotomy, IOL, and AUG.

Institutional review Board (IRB) approval was obtained (Approved by National Committee of Bio. & Med. Ethics with reference No 130-18). The study took into consideration ethical principles for conducting research, which includes beneficence, respect for human dignity, and justice. Because the study is a retrospective chart review study that used preexisting data, not all aspects of human subject protection may apply.

For analysis, we used descriptive statistics of number and percentage for reporting incidences of atonic PPH and for identifying the categorical study variables along with mean, median, and standard deviation to describe the continuous variables.

Regarding statistical inference, we categorized the amount of the blood loss in women who had atonic PPH during vaginal delivery into mild (500–1000 mL) and severe (>1000 mL) for the purpose of the study. Chi-square is used to determine the relationship between the severity amount of the blood loss and associated variables while the ordinal logistic regression model used to determine the risk factors associated with severity of blood loss in vaginal delivery. The null hypothesis measured the severity of blood loss with associated pairwise comparison, and the alternative hypothesis was blood loss with pairwise independent collection.

We used SPSS software (IBM, Chicago, IL, USA) to execute an ordinal logistic regression model. Blood loss (mild and severe) was used as outcome variable, and all others were independent variables. The regression model used the following equation: Blood Loss in Vaginal Birth = Age + Parity + Gestational Age + BMI + Epidural + Episiotomy + Iron Deficiency + IOL + AUG + Hemoglobin Level + Birth Weight.

## 3. Results

Of the 15,483 deliveries documented in the hospital data base, a total of 386 (2.5%) were atonic PPH. The total increasing rate from 2015 to 2018 was 1.87%. The increasing rate from 2016 to 2017 was 12.7%, and 2017 to 2018 was 14% Table 1. Out of the total atonic PPH cases, 220 (57%) cases were vaginal birth and 166 (43%) were CS birth.

Table 2 represents the total sample characteristics in regards to age, gestational age, BMI, and hemoglobin level at the last antenatal visit. The mean of the estimated blood loss for the total study sample was 1096.54 mL with a median of 1000 mL (SD = 722.53 mL). Out of the total sample, 125 (32.4%) had a history of CS, with 372 (96.4%) of the participants receiving a total of 20 units of Pitocin (to enhance uterine contractility).

Table 3 represents the characteristics of women who had a PPH diagnosis during vaginal birth. The average estimated amount of blood loss was 882.29 mL, with a median of 700 mL (SD = 576.85 mL). The majority of the participants received a total of 20 units of Pitocin during labor. Regarding episiotomy, 70 (31.8%) participants presented with episiotomy while only 9 (4.1%) experienced third or fourth perineal tears. Out of the total 220 PPH women, 67 (30.5%) were primipara (first pregnancy), 54 (24.5%) women were in their second pregnancy, 27 (12.3%) participants had IOL, and 41 (18.6%) had AUG. In all vaginal birth participants, the placenta was delivered spontaneously; only 21 (9.5%) had epidural anesthesia. None of the sample were exposed to instrumental birth.

Table 4 lists the association of each predictor with the response variable blood loss using a chi-square test. Findings revealed significate association between the severity of blood loss and epidural (*p* = 0.013), episiotomy (*p* = 0.038), IOL * epidural (*p* = 0.004), interaction of IOL * AUG * Epidural (*p* = 0.008) with insignificant association > 0.05 with age, parity, gestational age, BMI, hemoglobin level, Iron Deficiency anemia, and birth weight.

The findings showed significant association between blood loss and the epidural (F = 6.314, df = 1, *p* = 0.013), episiotomy (F = 4.38, df = 1, *p* = 0.038), and IOL (F = 1.224, df = 1, *p* = 0.004). The interaction between IOL, AUG, and an epidural on blood loss was significantly associated with the severity of atonic PPH (F = 7.24, df = 1, *p* = 0.041). Age, parity, gestational age, BMI, iron deficiency, hemoglobin level, and birth weight had *p*-values that exceeded 0.05, which reflected insignificant association with the amount of blood loss during vaginal birth Table 5.

## 4. Discussion

The findings of the study revealed a 2.5% incidence rate of atonic PPH in a tertiary hospital in Jeddah, Saudi Arabia. In addition, the findings showed a 1.8% increase in the rate of atonic PPH from 2015 to 2018, which is comparable with other studies in many countries that report increasing in the incidences of atonic PPH [14,15,16]. Mehrabadi et al. showed the rate increasing by 33% in British Columbia, while in Ireland the rate increased from 1% in 1999 to 3.4% in 2009 [15]. The increasing rate in the study sample was lower than the rate in British Colombia which could be explained by our study’s smaller sample size and one setting for our data collection. Moreover, as a university hospital not the whole population would have access to its care, only people who are working in academia, students, and university employees which limits population variation. Studies also showed repeated increasing rates each year except from 2015 to 2016 which showed a slight drop in the incidence of atonic PPH, this would be explained by the new policy that has been implemented: “baby friendly hospital”. However, the incidences returned to increase from 2016 to 2018 which is considered to align with other studies’ increasing rates [14,15]. The continuity of the increasing rate since 2016 could have many possible explanations, such an as increasing rate of CS at the study setting, an intensive use of medical interventions during the normal birthing process such as artificial rupture of membranes, higher dose of uterotonic medications [16,17,18,19,20]. Additionally, the ethnic background may be one of the explanations, as in the 2018 births around 9.1% of the sample were Yemeni; we did not report the nationality in our study because more than 60% of the sample were Saudi, however ethnic background could be one of the possible reasons [12].

Our analysis revealed that episiotomy is associated with increased severity of atonic PPH; women who had episiotomies were at higher risk of bleeding. This finding confirmed the results of earlier studies conducted in Spain and Saudi Arabia, which found that spontaneous perineal trauma or episiotomy is a risk factor for increasing PPH [21,22,23]. Additionally, 31% of the study sample had an episiotomy procedure, comparable to the 35% incidence rate of performing episiotomies in a tertiary hospital in Saudi Arabia [19]. Episiotomy is a common obstetric intervention in Saudi hospitals, and national efforts are required to reduce the procedure rate with rigorous follow-up to minimize complications [24,25].

Moreover, the study findings revealed a significant association between the severity of atonic PPH and IOL and AUG. Similarly, Ekin et al. (2015) showed a significant association of severe PPH and AUG, [21], and Sheldon et al. found significant association between severe atonic PPH and IOL [9]. Oxytocin is the most common medication used to induce and augment labor [26]. Additionally, Oxytocin is prescribed as an active management of the third stage of labor, with a recommend dosage of 10 IU [27]. However, several studies revealed a direct relationship between the amount of oxytocin administered and the incidences and severity of the atonic PPH [10,16,17,18,19]. This explains why women who undergo IOL and AUG using oxytocin have a higher risk of severe atonic PPH. This is due to exposure to a large amount of oxytocin compared to women who did not receive IOL or AUG, which is evident in the significant association of the interaction of induction and augmentation with severity of atonic PPH. Therefore, caution must be exercised with women who had IOL, AUG, or both in order to prevent the incidence of atonic PPH or decrease its severity.

Regarding epidurals’ effect on the severity of atonic PPH, our findings did show significant association between epidural alone and the severity of atonic PPH, which align with a previous study that stated that epidural is one of the risk factors of atonic PPH [14]. The association between epidural anesthesia and atonic PPH explained by the effects of epidural on the prolongation of the labor which leads to exhaustion of the uterine muscles and ends up with uterine atony. Moreover, the interaction between induction, augmentation, and epidural showed significant association with severe atonic PPH. This finding might be explained by the risk assessment of PPH, which relied on counting the number of risk factors that women had, correlated with the possibility of predicting the severity of atonic PPH [9,28].

Regarding parity and BMI, our study findings did not find significant association between the severity of atonic PPH and age and parity (number of pregnancies) in our sample; however, the majority of women who had atonic PPH were nulliparity (first pregnancy). In addition, the mean BMI was 29, which constitutes being overweight, according the international BMI index. These observations align with previous studies’ findings [9,16,29,30].

Regarding hemoglobin level, even though our analysis did not determine significant association of low hemoglobin level with severity of atonic PPH, the average hemoglobin level in our sample was 9.5, considered an abnormally low level. This observation is comparable with previous studies that have suggested that women with anemia at the time of birth were more likely to experience significant blood loss [9,29].

Our study has several strengths as well as limitations, the important strength being answering the international call reflected in the SDG target 3.1, to reduce global MMR to less than 70 in 100,000 by 2030 and to reduce the baseline rate of 2010 to 70% by 2030, which could not happen without a detailed investigation of the leading cause of obstetric hemorrhage, PPH. Another strength is being the first study to investigate the incidences and risk factors of atonic PPH in Saudi Arabia that addresses the findings of MMR in Saudi Arabia based on the WHO report in 2017. To ensure access to every case of atonic PPH and minimize the risk of under-reporting cases, we used two methods to access atonic PPH cases charts by using ICD codes, and manually by reviewing the labor and delivery registry books for 4 years back. The limitation of our study is that of any retrospective chart review that relies on previous documentations that might be inaccurate, incomplete and shown variation of healthcare providers documentation. Data about history of previous placental complications, medical history of the patients (e.g., hypertension, Diabetes.) and pregnancy-related complications such as preeclampsia, eclampsia or gestational diabetes were not collected, which may have added more information regarding atonic PPH cases in Saudi Arabia and added more risk factors. Moreover, the documentation of the duration of second and third stage of labor was not clear in the patient file and no clear answer was taken from the nurses in the labor room, so we remove the labor duration variable which added another limitation to the study. In addition, using atonic PPH data from one setting in Saudi Arabia limits the generalization of our findings regarding atonic PPH incidences and risk factors to the Saudi population in which we need to uncover the trends of atonic PPH.

## 5. Conclusions

In conclusion, the incidence of atonic PPH in the total sample was 2.5%, with the rate increasing by 12% between 2017 and 2018 in a tertiary hospital in Saudi Arabia. Risk factors that increase the severity of atonic PPH are epidural, episiotomy, induction of labor, and induction supplemented with augmentation of labor. Women who had an episiotomy, induction of labor, or augmentation are predicted to have a larger amount of blood loss (>1000 mL) during vaginal delivery compared to patients who did not receive those procedures. The clinical implications of the study would be the reevaluation of the postpartum hemorrhage risk assessment tool and considering epidural, induction of labor, augmentation of labor, and episiotomy as factors that might increases the risk scores. This will help for the early detection of risky women for severe atonic PPH, which minimizes the adverse effects of the disease and facilitates the early management of it. Future studies are needed regarding the national trends of atonic PPH in Saudi Arabia, with a larger sample size in different settings across the country. This could be national study to ensure a good representation of the Saudi population.

## Figures and Tables

**Table 1:** Description of the number of atonic postpartum hemorrhage (PPH) cases per year.

Year	Number of Total Deliveries	Frequancy of Atonic PPH Each Year	Perecentage %
2015	3553	92	2.6
2016	3997	87	2.2
2017	4321	106	2.5
2018	3612	101	2.8
Total	15,483	386	

**Table 2:** Total Sample Characteristics.

	Age	GA *	Birth Wt.	BMI	Hemoglobin
*N*	386	366	385	383	386
Missing	0	20	1	3	0
Mean	30.62	37.69	3.00	29.84	9.62
Std. Deviation	6.34	3.62	0.77	5.75	3.73

* GA= Gestational Age.

**Table 3:** Vaginal Birth Sample Characteristics.

	Age	GA *	Birth Wt.	BMI	Hemoglobin
*N*	220	205	220	217	220
Missing	0	15	0	3	0
Mean	28.90	38.36	3.11	29.15	9.57
Std. Deviation	6.51	3.44	0.68	5.44	1.71

* GA= Gestational Age.

**Table 4:** Relationship between Dependent (severity of atonic PPH) and Independent Variables.

Blood Loss	VS	*p*-Value
	Age	0.945
	Parity	0.727
	Gestational Age	0.153
	BMI	0.142
	Epidural	0.013
	Episiotomy	0.038
	Iron Deficiency anemia	0.627
	IOL	0.004
	IOL * AUG * Epidural	0.008
	Hemoglobin Level	0.347
	Birth Weight	0.806

* associated at 5%.

**Table 5:** Ordinal Logistic Regression Blood Loss as Dependent Variable.

Variable	F	df	*p*	Adj. R
Age	0.005	1	0.945	0.190
Parity	0.123	1	0.727	0.001
Gestational Age	2.058	1	0.153	0.012
BMI	2.178	1	0.142	0.013
Epidural *	6.314	1	0.013	0.190
Episiotomy *	4.375	1	0.038	0.048
Iron Deficiency	0.236	1	0.627	0.036
IOL *	1.224	1	0.004	0.048
IOL * AUG * Epidural *	7.236	1	0.008	0.041
Hemoglobin Level	0.891	1	0.347	0.005
Birth Weight	0.061	1	0.806	0.190

* significant at <0.05.
